# Benchmark Approach to Unravel Fluoride Toxicity: Liver and Kidney Disruptions in Subacutely Exposed Rats

**DOI:** 10.3390/jox16020063

**Published:** 2026-04-07

**Authors:** Jelena Radovanović, Sanja Milutinović-Smiljanić, Biljana Antonijević, Katarina Baralić, Marijana Ćurčić, Đurđica Marić, Zoran Mandinić

**Affiliations:** 1 Department of Radiobiology and Molecular Genetics, “Vinča” Institute of Nuclear Sciences-National Institute of the Republic of Serbia, University of Belgrade, 11000 Belgrade, Serbia; 2 Department of General and Oral Histology and Embryology, School of Dental Medicine, University of Belgrade, 11000 Beograd, Serbia; sanja.milutinovic@stomf.bg.ac.rs; 3 Department of Toxicology “Akademik Danilo Soldatović”, Toxicological Risk Assessment Center, Faculty of Pharmacy, University of Belgrade, Vojvode Stepe 450, 11221 Belgrade, Serbia; biljana.antonijevic@pharmacy.bg.ac.rs (B.A.); katarina.baralic@pharmacy.bg.ac.rs (K.B.); marijana.curcic@pharmacy.bg.ac.rs (M.Ć.); djurdjicamaric96@gmail.com (Đ.M.); 4 Clinic for Pediatric and Preventive Dentistry, School of Dental Medicine, University of Belgrade, 11000 Belgrade, Serbia; zoran.mandinic@stomf.bg.ac.rs

**Keywords:** fluoride exposure, benchmark modeling, bioelements, oxidative stress, hepatotoxicity, nephrotoxicity

## Abstract

The dose–response relationship for fluoride (F^−^) exposure remains largely unexplored. Hence, the current study assessed the hepatotoxic and nephrotoxic effects of subacute exposure (28 days) to increasing F^−^ concentrations in Wistar rats via the benchmark dose (BMD5) method. Thirty male rats were assigned to six groups (n = 5): a control group (tap water) along with five groups that received F^−^ via drinking water at increasing concentrations (10, 25, 50, 100, and 150 mg/L). F^−^ toxicity was determined via water intake, weight gain, histological analyses, redox status, and essential element levels. PROASTweb 70.1 software was utilized to investigate the external and internal F^−^ dose–response relationships. Specified major cytoarchitecture damage and superoxide anion (O_2_·^−^), total oxidative status (TOS), superoxide dismutase (SOD) activity, total thiol groups (SH), and advanced oxidation protein product (AOPP) level alterations were detected in both sets of tissues. Moreover, F^−^ caused an imbalance in copper (Cu), zinc (Zn), iron (Fe), and manganese (Mn). The most sensitive parameters were O_2_·^−^ (0.06 mg F^−^/kg) in the liver and AOPP (6.5 × 10^−6^ mg F^−^/L) in the kidneys. These findings contribute to the limited risk assessment of fluorides and highlight the dose-dependent relationship between redox status parameters and bioelements in the liver and kidneys.

## 1. Introduction

Even though fluorides (F^−^) have been used for more than 80 years for dental caries prevention [1,2], they remain one of the key focuses of research in modern toxicology. Given that F^−^ produce both positive and negative properties in human and animal physiology, their exposure levels and regulatory frameworks are continually evolving [3,4]. The Scientific Committee of the European Food Safety Authority (EFSA) is currently developing a new scientific opinion regarding F^−^ risk assessment in food and drinking water, which is expected to be published in 2025 [5].

Due to pesticide use and industrial pollution, F^−^ are found in food, water, air, and soil in high concentrations [6,7]. Additionally, they have an important role in the pharmaceutical industry, due to their influence on the bioactivity and metabolic stability of medications [8]. Nevertheless, excessive F^−^ intake can cause local and/or systemic toxicity [9]. To date, certain food groups in Europe have been evaluated, with high F^−^ concentrations identified in seafood and tree nuts [10,11]. Further studies are needed to better understand cumulative F^−^ intake from water, beverages, and other food groups in order to establish clear guidelines for the human population. Additional education about F^−^ use dedicated to professionals (dentists and oral hygienists) would also be beneficial for their safe application [12]. Moreover, understanding F^−^ metabolism is essential to prevent fluoride toxicity in human and animal populations. Approximately 50% of absorbed F^−^ are excreted via the urinary pathway within 24 h. However, renal clearance of F^−^ depends on individual physiological properties and environmental aspects. Urinary F^−^ excretion can be reduced due to various pH imbalances, which may result in F^−^ retention and possible toxicity [13,14]. Moreover, elevated F^−^ concentrations can impair any organ, especially the liver, the most important metabolic organ [15,16]. Indeed, different studies have highlighted the adverse effects of F^−^ in various species, both in vivo and in vitro [17,18,19]. Modern toxicology is advancing rapidly, and, since most toxicology studies are performed on animals, dose–response data are essential for identifying potentially hazardous endpoints of different substances. Dose–response modeling methods are especially valuable when statistical evidence is lacking, providing additional support for risk assessment [20]. Recently, the benchmark dose (BMD) approach has gained acceptance as a regulatory approved method for dose–response analysis. This method is considered a more advanced replacement for the no-observed-adverse-effect level (NOAEL) method. By utilizing a chemical dose–response approach, the BMD method estimates the potential extent of harm to organisms based on a specific level of exposure [21]. In contrast to NOAEL, BMD is derived through statistical modeling and accounts for variation in response rates to harmful effects [22].

Considering all of the above, the purpose of the current research was to evaluate the influence of subacute exposure to F^−^ via drinking water at five increasing concentrations on liver and kidney samples in Wistar rats by investigating changes in water consumption, bodyweight gain (BWG), redox status, histopathology, and essential element levels via the BMD approach. Based on our knowledge, this is one of the first studies to apply dose–response methods to assess F^−^ subacute toxicity in the liver and kidneys of experimental rats.

## 2. Materials and Methods

### 2.1. Chemicals

The chemicals utilized in the research were of analytical-grade purity. Sodium fluoride (NaF) was obtained from Kemika (Zagreb, Croatia), perchloric acid (HCIO_4_) from Fisher Scientific (Waltham, MA, USA), and nitric acid (HNO_3_) and chemicals for redox status and bioelement assessment from Sigma-Aldrich-Chemie (Steinheim, Germany). Formaldehyde was purchased from Boston BioProducts Inc. (Milford, MA, USA).

### 2.2. Animals and Experimental Procedure

The present research included 30 male albino Wistar rats, aged 8 weeks, with an average weight of 140–190 g, which were obtained from the Military Medical Academy (Belgrade, Serbia). The study was authorized by the Animal Experimentation Committee of the University of Belgrade, Faculty of Pharmacy (No. 323-07-11822/2018–05), and by the Ethical Committee of the School of Dental Medicine (Serbia, No. 36/2), in agreement with the ARRIVE guidelines, the Basel Declaration, the Guidance on the Operation of the Animals (Scientific Procedures) Act 1986 and EU Directive 2010/63 for the protection of animals used for scientific purposes. The animals were randomly selected (n = 5 per group, as a result of the 3R principle) and divided into 6 stainless-steel cages kept in a room with appropriate surroundings (temperature: 22 ± 2 °C, moisture: 60–70%, 12 h light/dark rotation). The use of five rats per group was determined using the Power Sample Size Calculation software (https://clincalc.com/stats/samplesize.aspx; accessed on 28 April 2025) and was in compliance with the OECD Test Guideline No. 407: Repeated Dose 28-Day Oral Toxicity Study in Rodents, which recommends a minimum of five animals per group [23]. Until the beginning of the experiment, the animals were given one week to acclimate. Food (The Veterinary Institute, Subotica, Serbia) and water were provided ad libitum. To mimic the most common route of F^−^ exposure, the rodents received F^−^ dissolved in drinking water, rather than via oral gavage. F^−^ solutions were kept in the refrigerator in plastic bottles and made daily. The control animals were given tap water, whereas the other five groups were given solutions made with tap water with increasing fluoride concentrations: 10, 25, 50, 100, and 150 mg/L NaF, for 28 days. In the present research, tap water was used for the control group. According to the National Guide of the Republic of Serbia, the maximum allowed concentration in tap water in Serbia is 1.2 mg/L, while it is reported that in some Belgrade municipalities, this level is much lower (0.153 ± 0.004 mg/L and 0.127 ± 0.003 mg/L) [24]. In the present study, the F^−^ amount in the tap water ingested by the animals (used in the control and to prepare all the test solutions) was measured and found to be 0.164 ± 0.0084 mg/L. Therefore, the low F^−^ levels naturally present in tap water did not affect data interpretation. The moderately elevated concentrations of F^−^ were selected in accordance with the literature data to perform BMD modeling [19,25,26,27]. A F^−^ concentration of 150 mg/L was selected because it is a well-established toxic dose in rats, shown to cause oxidative stress, DNA damage, and cellular dysfunction in previous studies and confirmed by our own preliminary data [28,29,30]. In this research, the mean F^−^ dose ingested by the rats was 19.2 mg/kg/day (calculated according to water consumption and body mass measurements), and doses between 31 and 102 mg/kg/day represent acute doses for F^−^ toxicity in rats [31]. Water consumption was evaluated each day for all the groups and daily water consumption was calculated to evaluate approximate exposure. According to body weight (measured on days 7, 14, 21 and 28) and average water consumption, the animals ingested 1.4, 3.5, 6.2, 13.6 and 19.2 mg F^−^/kg/day (external doses), which corresponded to subacute exposure. BWG was calculated using the following formula: (cbm-bbm)/bbm (cbm represents body mass at the measurement time point and bbm is the baseline body mass of each individual rat). After 28 days, euthanasia was performed by the intraperitoneal use of anesthetic xylazine (30 mg/kg) and ketamine (300 mg/kg).

### 2.3. Tissue Preparation

After euthanasia, tissue samples were extracted and splashed with 0.9% cold saline. Tissues were split for assessment of F^−^ tissue concentrations (i.e., internal doses), histopathological analyses, redox status parameters, and essential elements concentrations. Fragments were kept at −20 °C for F^−^ and essential element evaluation, or snap-frozen in liquid nitrogen and later preserved at −80 °C for redox status assessment. For histopathological analyses, liver and kidney tissues were stored for 48 h in 4% formaldehyde in 0.1 M phosphate-buffered saline (pH 7.4).

### 2.4. Histology and Histomorphometry

The specimens were cut (4 µm thick) with a microtome (Leica SM2000R; Leica Microsystems, Wetzlar, Germany), de-waxed, then routinely stained with hematoxylin and eosin (H&E). Three microscopic non-overlapping randomly chosen fields of view (40–400×) for every kidney and liver specimen were captured, utilizing a digital camera (Leica DFC295, Leica Microsystems GmbH, Wetzlar, Germany), and evaluated by Leica University Suite software (v4.3; Leica Microsystems, Germany). The histomorphometric analysis was carefully performed on liver [32] and kidney tissue [33].

Semi-quantitative analysis was performed on the rats’ livers utilizing the revised Knodell scoring system along with the histological activity index (HAI) to assess the levels of inflammation and necrosis. Accordingly, the main criteria regarding analysis were divided into portal inflammation, periportal and bridging necrosis, intralobular and focal necrosis, and fibrosis. For periportal and bridging necrosis, the rating scale was as follows: none (0), mild piecemeal necrosis (1), moderate piecemeal necrosis (involving less than <50% of the circumference of the majority of portal tracts) (3), marked piecemeal necrosis (involving less than 50% of the majority of portal tracts) (4), moderate piecemeal necrosis along with bridging necrosis (5), designated piecemeal necrosis along with bridging necrosis (6), and multilobular necrosis (10). For intralobular degeneration and focal necrosis, the grading score system indicated that the tissue seemed normal (0), mild (acidophilic bodies, ballooning degeneration, and/or scattered foci of hepatocellular necrosis in less than one-third of lobules or nodules, nuclear pyknosis, and arterial dilatation) (1), moderate (involvement of one-third to two-thirds of nodules or lobules) (3), or marked (involvement of more than two-thirds of nodules or lobules) (4). For portal inflammation, the rating scale was as follows: none (0), mild (sprinkling of inflammatory cells in less than one-third of portal tracts) (1), moderate (increased number of inflammatory cells in one-third to two-thirds of portal tracts) (3), and marked (concentrated arrangement of inflammatory cells in more than two-thirds of portal tracts) (4). The combination of all the criteria as a HAI yielded results that were graded by applying the following scoring system: without inflammation (0), minimal inflammation (1–4), mild inflammation (5–8), moderate inflammation (9–12), and marked inflammation (13–18) of liver tissue [32,34]. For kidney analysis, the scores were related to microscopic changes coherent with tubular necrosis: tubular cell necrosis, tubular lumen dilatation, vacuolization of tubular cells, intra-tubular cylinders, and interstitial fibrosis [33,35]. In the scoring system for renal histopathology: 0 indicates normal renal tissue, 0.5 indicates small focal damaged areas, 1 indicates less than <10% of cortical damaged zone, 2 indicates 10% to one-quarter of cortical damaged zone, 3 indicates one-quarter to three-quarters of cortical damaged zone, and 4 indicates more than three-quarters of cortical damaged zone. Histological evaluation was performed according to the EGTI scoring system [33,35]. This system is a scoring system for renal injury with four independent components: endothelial, glomerular, tubular, and interstitial. A sum of these scores was specified for each animal and employed to compare the groups. For the tubular component rating scale, the rating system was as follows: without damage (0); loss of brush border (BB) in less than one-quarter of tubular cells and preserved integrity of the basal membrane (1); loss of brush border (BB) in more than one-quarter of tubular cells and thickened basal membrane (2) along with inflammation cast formation and necrosis in less than 60% of tubular cells (3) or necrosis in more than 60% of tubular cells (4). For the endothelial component rating, no damage (0), endothelial swelling (1), endothelial disruption (2), or endothelial loss (3) was recorded. The glomerular scoring system was as follows: without damage (0), thickening of the Bowman capsule (1), glomerular retraction (2), and glomerular fibrosis (3). For tubular/interstitial component ratings, the following scoring system was used: no damage (0) or inflammation and hemorrhage in less than one-quarter of tissue (1) along with necrosis in less than one-quarter of tissue (2), necrosis in less than 60% of tissue (3), or necrosis in more than 60% of tubular and interstitial tissue (4).

### 2.5. Assessment of Internal Fluoride Doses

An ion-selective F^−^ electrode (ISE type 800-Consort; Belgium; pH meter: Iskra MA 5735) [36] was used to measure internal F^−^ concentrations (later referred to as internal F^−^ doses in the BMD response) in the examined tissues. For the method accuracy testing, F^−^ standard solutions (0.01 mg/L and 0.1 mg/L; Merck Millipore, Darmstadt, Germany) were applied. Specifically, into each cap of the diffusion cell, 0.5 mL of 1 mol/L NaOH in ethanol was inserted, after which all the caps were placed into a laboratory oven (SAUTER, Basel, Switzerland) and heated for 2 h at 55 °C. Due to evaporation, a thin layer of NaOH in ethanol was formed in the cap. Liver and kidney tissues (2–4 g) were placed into the diffusion cells, and afterward 5 mL of 40% AgClO_4_ (22.5 g Ag_2_O dissolved in 100 mL 60% HClO_4_) and 1.5 mL of 70% HClO_4_ were added to the samples. Diffusion cells were covered with caps and placed into the laboratory oven at 55 °C for 24 h. Throughout the diffusion, F^−^ reacted with the NaOH and NaF was formed. The thin coat from the cup was washed with deionized water (5 mL) and placed into polyethylene plates. Finally, the TISAB buffer solution (the same volume, pH 5–5.5 mL) was added, with constant mixing in a magnetic stirrer (Tehtnica, Slovenia). The potential of F^−^ was considered using the following formula: E (mV) = −log [F^−^], with a quantification limit of 0.0015 mg/kg F^−^, in addition to a detection limit of 0.0005 mg/kg F^−^.

### 2.6. Assessment of Redox Status Parameters

Liver and kidney fragments (approximate weight: 0.3–0.4 g) were homogenized in a glass cylinder in 0.1 mol/L phosphate buffer (pH 7.4, 1:9 ratio) with a T10 basic Ultra-Turrax homogenizer (IKA, Königswinter, Germany). For post-mitochondrial supernatant extraction, homogenates were centrifuged at 800× *g* for 10 min, then at 9500× *g* for 20 min (centrifuge: 5415 R; Eppendorf, Hamburg, Germany). Superoxide anions (O_2_·^−^), total oxidative status (TOS), superoxide dismutase (SOD) activity, total thiol (SH) groups, malondialdehyde (MDA), advanced oxidation protein product (AOPP) level and protein concentrations were determined. For O_2_·^−^, TOS, SOD, and AOPP determination, an ILAB 300 plus analyzer (Instrumentation Laboratory, Milan, Italy) was utilized, while for MDA and total SH group evaluation, a Nano UV/VIS spectrometer (BMG Labtech, Ortenberg, Germany) was applied. The methodological principles were thoroughly presented in a previously published paper [28].

### 2.7. Assessment of Essential Element Concentrations

For the determination of Cu, Zn, Fe, and Mn concentrations, approximately 500 mg of liver and kidney tissues was used (the measurements were performed via an analytical balance (Radwag, Radom, Poland)). The fragments were cleaned with distilled and deionized water and placed into Erlenmeyer glass flasks (previously soaked with 10% HNO_3_). Then, 8 mL of 69% HNO_3_ and 2 mL of 71% HCIO_4_ were added for the digestion process. Specimens remained heated in a sand bath (Elektron, Trstenik, Serbia) at 200 °C. Determination of essential elements was performed by flame atomic absorption spectrophotometry (FAAS) (240FS AA; Agilent Technologies Santa Clara, CA, USA). Calibration was achieved with the ICM-100 calibration standard solution (Agilent Technologies) in the concentrations: 0.10 mg/L, 0.20 mg/L, 0.50 mg/L, 1 mg/L, 5 mg/L, and 10 mg/L. 1577c—Bovine liver (LGS Standard, Teddington, Middlesex, UK) and whole blood Level 2 (SeronormTM, Sero, Billingstad, Norway) were used as reference materials. The coefficient of variation (CV) for all analyzed bioelements was less than 5%. The limits of detection for Cu, Zn, Fe, and Mn were 0.003 mg/L, 0.002 mg/L, 0.050 mg/L, and 0.005 mg/L, respectively. The limit of quantification values for Cu, Zn, Fe, and Mn were 0.010 mg/L, 0.007 mg/L, 0.170 mg/L, and 0.017 mg/L, respectively.

### 2.8. Benchmark Dose Modeling

PROASTweb 70.1 software (Dutch National Institute for Public Health and the Environment (RIVM), https://proastweb.rivm.nl/, accessed on 20 March 2025) was utilized for benchmark dose–response modeling. External (concentrations of the solutions consumed by the animals via drinking water) and internal F^−^ dosages (F^−^ concentrations measured in tissue) were examined for all the investigated redox status parameters and essential elements. External dosages (fluoride in drinking solutions) reflected the exposure, while internal dosages (fluoride in tissues) indicated the actual biologically effective dose, accounting for absorption, metabolism, and individual variability, ensuring that both administered and effective exposures were considered in the dose–response analysis. Based on a recommendation by the EFSA [21], a benchmark response (BMR) of 5% at a 90% confidence level was used for continuous individual features (exponential and Hill family models) if the Akaike information criterion was fulfilled and the BMD5/BMDL5 ratio was lower than 10. BMR characterizes the size of the effect, while BMD5 characterizes the dose that triggers an alteration in the specific variable. The benchmark dose interval is defined by the BMDL5 (BMD lower) and BMDU5 (BMD upper) interval [37].

### 2.9. Statistical Analysis

Statistical analyses and graphs were achieved via GraphPad Prism 8 software (GraphPad Software, Inc., San Diego, CA, USA). The normality of the distribution for the examined variables was evaluated via the Shapiro–Wilk test. One-way ANOVA with Fisher’s least significant difference (LSD) post hoc test was utilized for normally distributed data (mean ± SD), while the Kruskal–Wallis test with Dunn’s post hoc test was applied for non-normally distributed data (median and range). Repeated-measures data were analyzed using linear mixed models (LMMs), while multiple testing was controlled by the false discovery rate (FDR). Correlation analyses for F^−^ external doses and internal doses were accessed by Pearson correlation for normal distributions and Spearman correlation for non-normal distributions. External F^−^ doses refer to the fluoride concentrations in the drinking water solutions, while external F^−^ concentrations represent the doses ingested by the experimental animals (calculations based on average water consumption and body weight). In contrast, F^−^ internal doses reflect the fluoride concentrations in the tissues. A *p*-value of <0.05 was considered statistically meaningful.

## 3. Results

### 3.1. Water Consumption and Body Weight Gain (BWG)

**Figure 1:**
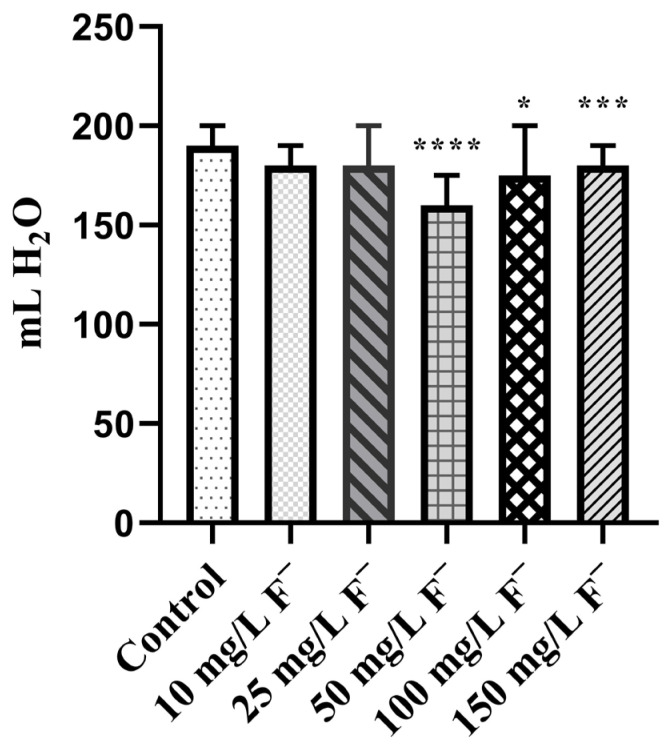
Average water consumption during 28 days among all experimental groups. Friedman test followed by Dunn’s multiple comparisons test. * *p* < 0.05; *** *p* < 0.001; **** *p* < 0.0001 (compared with the control group). Means and ± SDs.

The average water consumption during 28 days is presented in Figure 1. In the 50, 100, and 150 mg/L F^−^ groups, water intake was significantly reduced (* *p* < 0.05; *** *p* < 0.001; **** *p* < 0.0001) compared to the control. Table 1 displays the alteration in BWG, and a significantly increased BWG was observed within each experimental group by the 28th day in comparison with the 7th and 14th days of the experiment. BWG% was decreased in the 50, 100 and 150 mg/L F^−^ groups in comparison with the control by the end of the experiment.

### 3.2. Histologic and Histomorphometric Analysis

**Figure 2:**
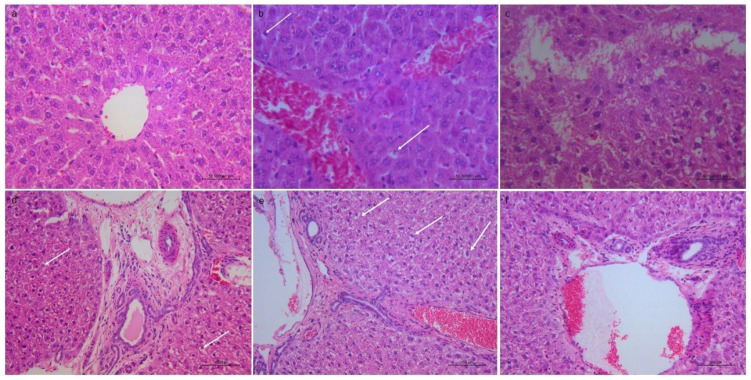
Representative micrographs of the liver tissue (H&E): (**a**) control group without pathological changes, 400×; (**b**) 50 mg/L F^−^, congested blood vessels, apoptotic hepatocytes with cytoplasmic and nuclear condensation (arrows), 400×; (**c**) 100 mg/L F^−^, focal necrosis, 400×; (**d**) 100 mg/L F^−^, portal–portal bridging necrosis, apoptotic hepatocytes, 200×; (**e**) 150 mg/L F^−^, portal–central bridging necrosis, apoptosis of hepatocytes with cytoplasmic and nuclear condensation and nuclear fragmentation 200×; (**f**) 150 mg/L F^−^, slight portal fibrosis, 200×.

**Figure 3:**
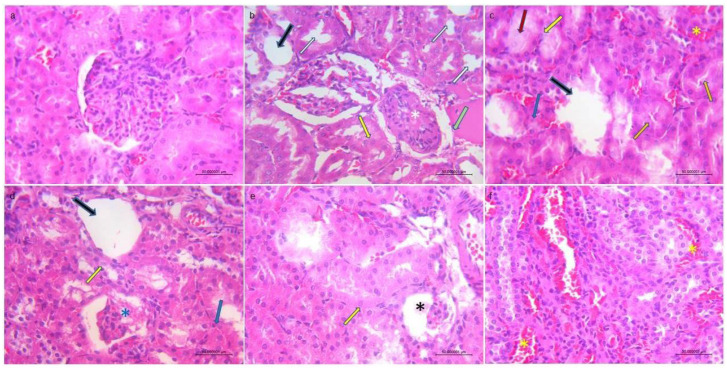
Representative micrographs of the kidney tissue (H&E): (**a**) control group without pathological changes, 400×; (**b**) 50 mg/L F^−^, 400×; (**c**) 100 mg/L F^−^, 400×; (**d**) 150 mg/L F^−^ 400×; (**e**) 150 mg/L F^−^, 400×; (**f**) 150 mg/L F^−^, 400×. White arrows—widened lumens and increased size of the tubular epithelial cells, yellow arrows—karyolisis, blue arrows—pyknosis, green arrow—thickening of Bowman’s capsule, black arrows—atrophic glomeruli, orange arrows—tubular cells are swollen and without brush border, red arrow—proximal tubule with epithelial necrosis and obliterated lumen, white *—partial hyalinization of glomeruli, blue *—retraction of capillary tuft, black *—glomerular atrophy, yellow *—congested vessel engaged with blood.

Progression of liver damage was signified by increased vacuolar degeneration of hepatocytes (leading to magnified hepatocellular size) together with disfigured nuclear dimensions (indicative of hepatocellular necrosis) (Figures S3–S5). Degenerative changes were also observed, as well as dilated and congested blood sinusoids and central veins (Figure S2). Infiltrates of inflammatory cells in the portal and intra-acinar parenchyma were consistent with hepatic injury (Figure S6). With a higher F^−^ dose, liver architecture was more severely damaged (Figure 2). In Table 2, the Knoddel scores for the livers are presented. Extensive damage in kidney tissue was observed in the groups exposed to 50 mg/L F^−^ and higher doses (Figure 3). Kidney injury was confirmed with degeneration of the tubular epithelial cells, congested vessels, atrophic glomeruli, karyolisis and pyknosis (Figures S8–S12). Table 3 summarizes the histopathological analysis and scoring of kidney cortical damage.

### 3.3. Fluoride Internal Doses

**Figure 4:**
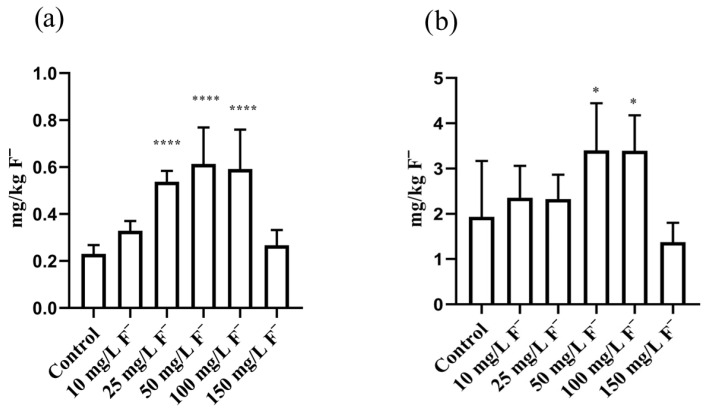
Liver (**a**) and kidney (**b**) fluoride internal doses (n = 5). One-way ANOVA followed by Fisher’s least significant difference (LSD) test. * *p* < 0.05; **** *p* < 0.0001 (compared with the control group). Results are presented as means and ±SDs.

Significantly higher F^−^ internal doses in the liver tissue were detected between the 25, 50, and 100 mg/L F^−^ groups (**** *p* < 0.0001, Figure 4a), with the highest mean value observed in the 50 mg/L group (0.61 ± 0.15 mg F^−^/kg). As far as kidney tissue is concerned, significantly increased F^−^ internal doses were observed in the 50 and 100 mg/L F^−^ groups (* *p* < 0.05, Figure 4b), with the highest mean value observed in the 50 mg/L group (3.40 ± 0.93 mg F^−^/kg). No correlations were observed between external F^−^ concentrations and internal doses, or between external F^−^ doses and internal doses, in either the liver or kidneys (liver: R = 0.7173, *p* = 0.109 and R = 0.7201, *p* = 0.107; kidneys: R = −0.1649, *p* = 0.755 and R = −0.1429, *p* = 0.787).

### 3.4. Oxidative Stress Analysis

**Figure 5:**
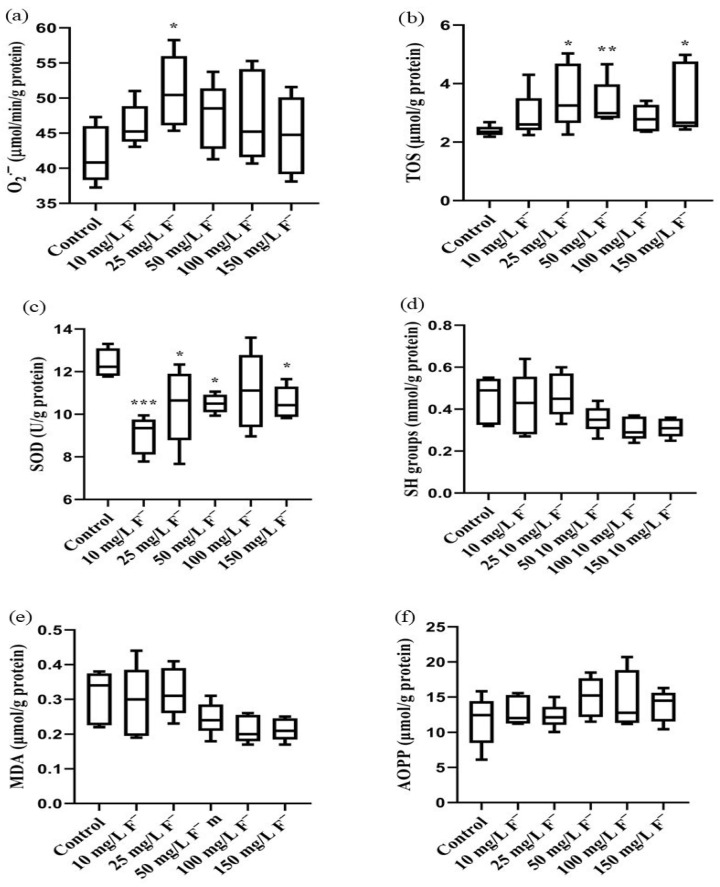
Impact of increasing fluoride concentrations (10, 25, 50, 100, and 150 mg/L F^−^, n = 5) on parameters of redox status in the livers of Wistar rats. (**a**) O_2_·^−^ superoxide anions (μmol/min/g protein); (**b**) TOS total oxidative status (μmol/g protein); (**c**) SOD superoxide dismutase activity (U/g protein); (**d**) SH total thiol groups (mmol/g protein); (**e**) MDA malondialdehyde (μmol/g protein); (**f**) AOPP advanced oxidation protein products (μmol/g protein). One-way ANOVA followed by Fisher’s least significant difference (LSD) and Kruskal–Wallis tests, followed by Dunn’s post hoc test. * *p* < 0.05; ** *p* < 0.01; *** *p* < 0.001 (compared with the control group). The lines inside the boxes represent the medians, the boxes represent the interquartile ranges (25–75%), and the whiskers represent the minimum and maximum values.

The prooxidative biomarker O_2_·^−^ was increased in the livers of all the treated groups, with an increase in the 25 mg/L F^−^ group (* *p* < 0.05, Figure 5a) compared to the control. TOS values were elevated in all the groups, significantly in the 25, 50, and 150 mg/L F^−^ groups (* *p* < 0.05; ** *p* < 0.01, Figure 5b) when compared with the control. Additionally, antioxidative biomarkers, SOD and SH groups were decreased across all the groups (Figure 5c,d), with SOD activity significantly reduced (* *p* < 0.05; *** *p* < 0.001) compared to the control. A decreasing trend in MDA and an increasing trend in AOPP values were detected in the higher-dose groups (Figure 5e,f), although without statistical significance when related to the control.

**Figure 6:**
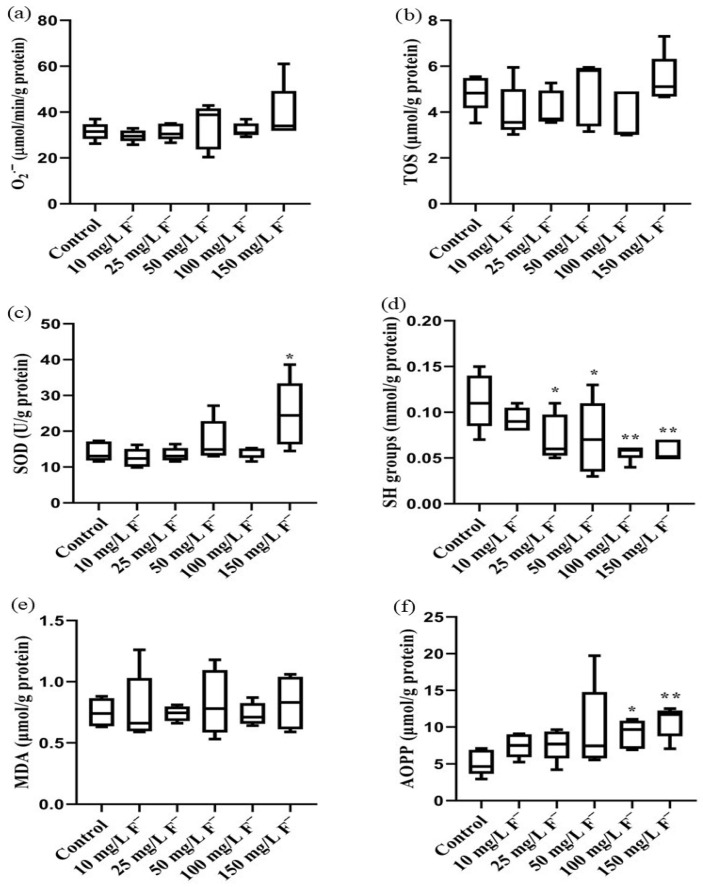
Impact of increasing fluoride concentrations (10, 25, 50, 100, and 150 mg/L F^−^, n = 5) on parameters of redox status in the kidneys of Wistar rats. (**a**) O_2_·^−^ superoxide anions (μmol/min/g protein); (**b**) TOS total oxidative status (μmol/g protein); (**c**) SOD superoxide dismutase activity (U/g protein); (**d**) SH total thiol groups (mmol/g protein); (**e**) MDA malondialdehyde (μmol/g protein); (**f**) AOPP advanced oxidation protein products (μmol/g protein). One-way ANOVA followed by Fisher’s least significant difference (LSD) and Kruskal–Wallis tests, followed by Dunn’s post hoc test. * *p* < 0.05; ** *p* < 0.01 (compared with the control group). The lines inside the boxes represent the medians, the boxes represent the interquartile ranges (25–75%), and the whiskers represent the minimum and maximum values.

In kidney tissue, although no statistical significance was observed, O_2_·^−^ and TOS were slightly increased in the highest-dose group (Figure 6a,b). SOD activity was significantly high in the 150 mg/L F^−^ group (* *p* < 0.05, Figure 6c), while SH group values were significantly decreased in the 25, 50, 100 and 150 mg/L F^−^ groups (* *p* < 0.05, ** *p* < 0.01; Figure 6c,d). MDA values were similar across all the groups. AOPP was elevated in all dose groups, with a significant increase observed in the 100 and 150 mg/L F^−^ groups (* *p* < 0.05, ** *p* < 0.01; Figure 6e,f).

### 3.5. Essential Metal Analyses

Cu levels in the liver were decreased with statistical significance in the 100 and 150 mg/L F^−^ groups (* *p* < 0.05, ** *p* < 0.01; Table 4). On the contrary, Zn levels were increased in all groups, while this change was significant in the 50 and 100 mg/L F^−^ groups compared to the control (* *p* < 0.05; ** *p* < 0.01). Similar observations were recorded for Mn, where statistically elevated levels were detected in the 100 and 150 mg/L F^−^ groups (* *p* < 0.05). As far as Fe is concerned, no statistically significant difference was observed. Similarly, in the kidney samples, a trend of decreasing Cu levels was detected (without statistical significance). Further, a trend of increasing Zn levels in the kidneys, with significance only in the 100 mg/L F^−^ group (* *p* < 0.05, Table 4), was observed. Finally, Fe and Mn levels were reduced, with statistical significance observed in the 50, 100 and 150 mg/L groups (* *p* < 0.05; ** *p* < 0.01; *** *p* < 0.001; **** *p* < 0.0001).

### 3.6. Benchmark Dose–Response Modelling

BMD5 analysis was conducted for all redox status parameters and essential elements measured in our study. Table 5 and Table 6 present the parameters that passed the criteria for dose–response modeling. In the liver tissues (Table 5), external dose-dependent effects were observed for SH groups, MDA, Cu, Zn and Mn. For SH groups and MDA, dose–response effects with decreasing trends were observed. Further, external dose-dependent effects with decreasing trends were established for Cu values, while external dose-dependent effects with increasing trends were established for Zn and Mn. An internal dose-dependent effect with an increasing trend was confirmed for O_2_·^−^. Considering external dose–response modeling, the lowest calculated BMDL5 in the liver was 0.1 mg F^−^/L for Mn, while for internal dose–response modeling, the lowest calculated BMDL5 was 0.06 mg F^−^/kg for O_2_·^−^.

In the kidney tissue (Table 6), external dose-dependent effects were observed for SOD activity, SH groups, AOPP, Cu, Zn, Fe, and Mn. Increasing dose–response trends were confirmed for SOD activity and AOPP. Additionally, a decreasing dose-dependent trend was observed for SH group values. A decreasing dose-dependent trend was also seen for all essential elements. Interestingly, internal dose-dependent trends showed a decrease for SOD activity and an increase for Cu and Zn values. Considering external dose–response modeling, the lowest calculated BMDL5 in the kidneys was 6.5 × 10^−6^ mg F^−^/L for AOPP, while for internal dose–response modeling, the lowest calculated BMDL was 1 × 10^−4^ mg F^−^/kg for SOD activity.

## 4. Discussion

Our in vivo study has demonstrated that moderate F^−^ exposure through drinking water reduces water consumption and BWG, disrupts cytoarchitecture, induces oxidative stress (OS), and disrupts the balance of essential elements in the liver and kidney tissues of Wistar rats. Additionally, the subacute external and internal dose–response of F^−^ in these tissues was confirmed using BMD5 modeling.

Given that fluoride exposure is widespread—from prophylactic agents in dentistry, natural sources, food, air, industrial pollution, and water fluoridation—local and/or systemic toxicity is a real concern [38]. As drinking water is the primary route of F^−^ exposure, most research, including ours, has focused on this source. However, comprehensive studies encompassing different exposure sources are needed for accurate cumulative F^−^ intake assessment. Monitoring F^−^ consumption from varied sources may optimize their use in pediatric dentistry and help prevent chronic toxicity. Since F^−^ are some of the most reactive elements, in the case of an excessive amount in the body, F^−^ affect oxygen, resulting in the production of hydrogen peroxide [39]. Indeed, many authors have recognized the significance of analysis related to F^−^ toxicity, since F^−^ toxicity exerts various cellular effects, and the main interactions are related to enzymes [40]. However, these animal studies were mainly concentrated on a maximum of three doses [19,26,41]. Additionally, liver- and kidney-related dose–response effects were not evaluated in the aforementioned studies. Data on the BMD5 modeling for F^−^ are available mainly for human urine samples, though they do not include the exact and specific time and dose of exposure [42,43].

Fluoride exposition is related to reproductive anomalies in both females and males, resulting in apoptosis, DNA damage, OS, hormonal imbalance, ovarian injury, reduced spermatogenesis, and sperm quality [44,45,46]. Thus, in our previous study, we addressed BMD5 modeling for increasing F^−^ concentrations regarding testicular toxicity [28]. Currently, there is a lack of data on the BMD5 for the potential toxic influence of F^−^ on the liver and kidneys, highlighting the urgent need for updated risk assessments. Our experimental study evaluated subacute F^−^ toxicity at five moderately increased concentrations in drinking water, BWG, histopathological architecture, redox status parameters, and essential elements in liver and kidney tissues of rats. Additionally, BMD5 response modelling was used for establishment of the dose intervals linked to a specific change in the investigated parameters.

In the current experiment, water intake was reduced across all the experimental groups, with a significant decrease observed in those exposed to F^−^ concentrations of 50 mg/L or higher. Regarding BWG, increased individual values were observed among all experimental groups by the end of the experiment (28th day), with the greatest statistical significance in comparison with the 7th and 14th days of the experiment. Based on the literature, F^−^ have been shown to reduce BWG [47,48], which finding is comparable with the results of our research, where the most pronounced reduction in % of BWG in comparison with the control was confirmed in the groups consuming 50 mg/L F^−^ and higher by the end of the experiment. As expected, the F^−^ internal doses in the liver and kidneys were significantly elevated, and accumulation was confirmed in the 25, 50, and 100 mg/L F^−^ groups. Interestingly, internal doses were similar to those of the control in the 150 mg/L F^−^ group. This may be explained by a potential saturation effect, whereby at higher F^−^ concentrations, the organism reaches a threshold beyond which further accumulation of F^−^ becomes limited. In animal studies, the dose–response relationship is a result of toxicodynamic (the interaction of the tested substance with specific cells/tissues) and toxicokinetic (absorption, metabolism, distribution, and excretion) processes [49]. The saturation of toxicokinetics in our research led to no further increase in F^−^ internal doses in either set of tissues, regardless of high F^−^ drinking concentrations. Thus, the use of computational modeling (such as BMD) is desirable, especially in situations where a saturation effect is present, in order to identify exposure doses of potentially toxic substances. The threshold and saturation effects observed in our dose–response study represent a non-linear response, and it is recommended that the internal dose should be utilized for a better understanding of the influence of toxicokinetic non-linearity on the dose–response relationship [49]. Bearing in mind that the kidneys and liver are the primary organs for F^−^ detoxification, we assume that some metabolic enzymes and/or membrane transporters became “overfilled” such that additional transport from the tissue is prevented. In our research, the resulting hepatotoxicity and nephrotoxicity are driven by oxidative stress and histopathological alterations, leading to several forms of cellular damage.

According to the histological findings, the most prominent damage in the liver tissue was recorded in the 50, 100 and 150 mg/L F^−^ groups (Figures S2–S6), whereas the control group showed no pathological changes (Figure S1). In these groups, after 28 days, pyknotic nuclei, inflammatory cells, dilatation of sinusoids and central veins, necrosis, and fibrosis were confirmed. Based on Knodell’s scores, the most pronounced liver injury-marked inflammation was noted in the group receiving 150 mg/L F^−^. The abovementioned results are consistent with prior research, which showed that exposure to 12 and 100 mg/kg F^−^ in Swiss male and Wistar albino rats caused congestion and hemorrhage as well as degeneration and necrosis of parenchymal cells in hepato-renal tissues, which was expected, as the liver performs a key function in xenobiotic detoxification [50,51]. Chronic exposure to fluoridated drinking water (concentrations of 8 ppm or greater) caused renal functional and structural changes, including tubular necrosis, atrophy, swelling and degeneration of tubular epithelium [39,52]. In our study, the control group showed no pathological changes (Figure S7), mild kidney damage was noted in the 25 mg/L F^−^ group, while in the groups exposed to higher concentrations, the impairments were more severe, with vacuolar degeneration, atrophy, glomeruli necrosis and nuclei abnormalities being observed (Figures S8–S12). Further, a novel scoring method (EGTI) for acute kidney injury showed endothelial, glomerular, tubular, and interstitial damage to be prominent in the groups exposed to higher doses. In studies regarding fluoride nephrotoxicity, the use of this scoring method has been limited, though in one recent study, acute tubular injury was confirmed in detail via EGTI scoring in Wistar rats that were exposed to water samples with a high F^−^ content [53].

Understanding of the intracellular pathways that participate in the cell–fluoride interaction is partial due to the variety of the molecular events [40]. However, as significant conclusions, studies have pointed out that excessive F^−^ could impair antioxidant capacity and cause OS, apoptosis, autophagy, inflammation and mitochondrial damage, particularly mitochondrial respiration, through the NADH oxidative respiratory chain, leading to reduced ATP production in various species and tissues [15,44,54,55]. Mitochondria represent the main source of reactive oxygen species (ROS) production, and F^−^ toxicity is associated with ROS induction, the generation of nitric oxide, and the reduction of cellular antioxidant defenses versus oxidative damage [40]. In addition to calcified tissues, the literature data are mainly focused on adverse impacts on liver, kidney and reproductive functions [15,44,56,57]. It was confirmed that OS caused by F^−^ in hepatic and renal cells could damage all cellular components and antioxidant defenses, triggering inflammation, protein oxidation, lipid peroxidation and comprehensive toxicity [15,58]. Accordingly, we evaluated a dose–response F^−^ dependence for a precise critical effect of several redox status parameters. O_2_·^−^ can be detected in almost all cells after damage and represent an important class of radicals for mitochondrial respiration [59]. In our research, in the liver we noted a tendency for elevated O_2_·^−^ values in all dose groups, with statistical significance noted in the 25 mg/L F^−^ group. In kidney tissue, a tendency for elevated O_2_·^−^ levels was also observed in the highest-dose group. TOS, representing systemic OS, was also evaluated in our study and was significantly elevated in the liver in the higher-dose groups. Taking all this together, it can be concluded that F^−^ alter prooxidative biomarkers in the investigated tissues. Corresponding to the BMD5 analyses, an internal dose–response relation among F^−^ and O_2_**·**^−^ in the liver has been confirmed with the BMDL5 of 0.06 mg F^−^/kg for O_2_**·**^−^. In the kidney tissues, O_2_·^−^ and TOS values were elevated in the highest-dose group, but without statistical significance or a dose–response relationship being confirmed. SOD is an important part of the endogenous antioxidative protection system which can be detected in the cytoplasm, nuclear components, and lysosomes [60]. Decreased SOD activity has been noted in various tissues in animal studies after exposure to elevated F^−^ doses, including the liver [61,62]. According to our results, SOD activity in the liver was significantly decreased in all groups, mainly in the 10 mg/L F^−^ group. However, the less pronounced decrease in SOD activity in the higher-dose groups may reflect an adaptive response to mitigate oxidative stress. Further, we noticed a trend of decreased values in higher-dose groups for another antioxidant biomarker—SH groups. Total thiol groups are important for the removal of ROS, and their reduced levels could contribute to the development of OS [63]. In the kidneys, significantly reduced SH groups were recorded in all dose groups. Notably, the external dose–response relationship between F^−^ and decreased SH groups was confirmed for both the liver and kidneys. The BMDL5 for SH groups in the liver was 10.8 mg F^−^/L, and for the kidneys it was 0.002 mg F^−^/L, suggesting that even such low doses may lead to 5% alterations in effects compared to the control. Interestingly, in the kidneys, we observed increased SOD activity in the 150 mg/L F^−^ group. The decrease in SOD levels in the liver across all fluoride-exposed groups suggests impaired antioxidant defense, while the increase in the kidneys at 150 mg/L may reflect compensatory upregulation in response to higher oxidative stress. The external dose–response relationship with an increasing trend for F^−^ and SOD in the kidneys was confirmed with the BMDL5 of 42.1 mg F^−^/L. For more accurate analyses of redox status after F^−^ exposure, we also evaluated oxidative damage biomarkers, MDA and AOPP. In cell membranes, MDA is often used as a marker of lipid peroxidation, and it has been shown that F^−^ alters its values [44,64]. However, interesting results were observed regarding MDA in the liver. A trend of decreased MDA values was noted in the groups that received F^−^, while BMD5 analysis showed a dose-dependent correlation. This may suggest that higher fluoride concentrations could trigger mechanisms that reduce lipid peroxidation, possibly through increased antioxidant defense or metabolic adjustments. The BMDL5 for MDA in the liver was 9.6 mg F^−^/L. Free radicals can also impact proteins, and their oxidative alterations can be presented through AOPP levels [65]. Some authors have pointed out that AOPP is an adequate biomarker for OS occurrence in the liver and kidney tissues, but still there are not enough data regarding the influence of F^−^ on AOPP values [66,67,68,69]. We observed an increasing trend for AOPP values in the liver in the higher-concentration groups. Additionally, in the kidneys, statistically elevated AOPP values were confirmed in the higher-concentration groups, and these findings were validated by external dose–response correlations between F^−^ and increased AOPP values. The BMDL5 for elevated AOPP values in the kidneys was 6.5 × 10^−6^ mg F^−^/L These results relate to the involvement of OS in the metabolic disturbances caused by F^−^. The complexity of F^−^ toxicity is obviously closely associated with dose and concentration (hormesis effect). There are still many questions regarding the influence of F^−^ on mitophagy and mitochondrial organization, growth, and composition [70]. According to the literature data, fluorides are linked to different molecular mechanisms and have the ability to alter numerous signaling pathways, including mitogen-activated protein kinase (MAPK), nuclear factor kappa B (NF-κB), and tumor protein p53 (P53) [54,71,72]. Further, fluorides stimulate apoptosis via two significant signaling pathways—mitochondrial-mediated and endoplasmic reticulum stress pathways [73]. These pathways are related to programmed cell death, cellular stress responses, and cancer-associated signaling, suggesting a possible connection between F^−^ toxicity and various diseases. Future studies should include in silico toxicogenomic tools with the aim of identifying key gene biomarkers involved in F^−^ toxicity. Finally, our research highlighted that increased F^−^ concentrations lead to pro- and antioxidant imbalance, and further research perhaps should also be linked to nonenzymatic antioxidants (glutathione, carotenoids, and flavonoids). Nevertheless, despite controversial results reported in the literature and the results from our study, F^−^ should be considered potential toxic compounds in the field of toxicology and epidemiologic/ecological research [40].

The alteration of metabolic pathways is associated with hepatic energy metabolism, and elevated F^−^ concentrations are also responsible for these alterations, affecting hepatic proteins [74]. Fluoride could impact renal functions, increasing levels of different essential bioelements and preventing the kidneys from removing toxic substances [74]. Acidic conditions could notably escalate F^−^ retention in the body and consequently could lead to toxicity and cell stress [75]. Fluoride distribution in the body deeply depends on pH conditions, and in an acidic environment hydrogen fluoride is formed, which passes through cell membranes easily due to its ionic form. Hydrogen fluoride is a small, lipid-soluble molecule, and its accelerated penetration into cells contributes to F^−^ reabsorption from the renal tubule into the circulation. Additionally, reduced renal clearance leads to F^−^ accumulation in soft and calcified tissues [40,76]. Ultimately, low pH enables additional F^−^ to pass into the cell, causing cellular damage [77]. Thus, we also evaluated the influence of F^−^ on essential elements (Cu, Zn, Fe, and Mn) in the liver and kidney samples of the experimental rats to evaluate how impaired functioning of these tissues affects some key nutrients. These bioelements are present in various tissues and are essential for organism homeostasis, mainly due to their antioxidative features. Cu and Zn are present in SOD1 and Mn in SOD2, which is important considering that, in our research, we evaluated total SOD (comprising both SOD1 and SOD2) [78,79,80,81]. Furthermore, bioelements act as cofactors in numerous physiological processes essential for cellular homeostasis and survival, as well as for the development of organs and tissues [82]. Data related to the impact of F^−^ on bioelement balance are scarce [83], and there are many reasons for mutual interactions between F^−^ and bioelements [84]. Cu, Zn, Fe, and Mn are essential for oral health, since they are closely related to tooth remineralization, and possess anti-decay and anti-bacterial features [85,86,87]. Considering that bioelements in excess or deficit could cause various disorders, their levels should be balanced. Based on our information, this is the first study to examine the influence of five increasing F^−^ concentrations on Cu, Zn, Fe and Mn levels in liver and kidney tissues after subacute exposure of rats through drinking water. Cu is widely used in dental materials, mainly for caries prevention, and its interaction with F^−^ is important to evaluate [88]. In our experiment, the presented values of essential elements were obtained from wet-organ weights. In the liver, we observed a significant reduction in Cu levels in the high-dose groups, while in the kidneys a trend of decreasing values was noted. An external dose–response relationship was confirmed for decreased Cu in the liver, and the BMDL was 30.1 mg F^−^/L. In the kidneys, an external dose–response relationship was also confirmed for decreased Cu levels, and the BMDL5 was 28.1 mg F^−^/L. These findings align with a study on pigs, which suggested that excess fluoride reduces copper levels in both the liver and kidney cortex [89]. A decrease in Cu could affect important biological processes in the liver and kidneys and even disrupt physiological functioning. A lack of Cu could affect Fe transport and hemoglobin synthesis [90], consequently leading to multiple disorders, including oral pathologies [91,92]. In our experiment, a trend of decreasing Fe levels in the high-dose groups was observed in the liver, while in the kidneys we confirmed a significant Fe reduction in groups exposed to 50 mg/L F^−^ and higher doses. This Fe reduction in the kidneys was confirmed via an external dose–response relationship, and the BMDL5 was 7.4 mg F^−^/L. Zn balance in the oral cavity is crucial since it participates in tooth development and is often used in anti-caries agents, such as mouth rinses and toothpastes [93,94]. In the present study, slight elevations of Zn levels in the liver and kidneys were detected in groups treated with moderately high doses and significantly increased in groups treated with higher doses. An external dose–response relationship was confirmed for increased Zn levels in the liver, and the BMDL5 was 7.3 mg F^−^/L. In the kidneys, an external dose–response relationship was confirmed for increased Zn levels, and the BMDL5 was 47.8 mg F^−^/L. We assume that Zn and F^−^ showed potential additive/synergistic effects in the groups with higher F^−^ doses. Data regarding interactions between F^−^ are Mn are extremely limited. In an experimental study performed on mice, excessive F^−^ disturbed Mn metabolism in the liver and kidneys [95]. Our findings are similar, since Mn levels were significantly elevated in the liver in the groups that received higher F^−^ concentrations, suggesting that F^−^ exposure might lead to an increase in manganese in the liver. On the other hand, in the kidneys, the observations were quite the opposite—reduced Mn levels, possibly indicating a disruption in the renal handling or distribution of manganese due to fluoride exposure. These results were confirmed through the BMD methodology, and an external dose–response relationship was confirmed for increased Mn levels in the liver, and the BMDL5 was 0.1 mg F^−^/L, while in the kidneys an external dose–response relationship for decreased Mn levels was confirmed, and the BMDL5 was 83.1 mg F^−^/L. Considering water fluoridation, F^−^ are commonly combined with other elements, and synergistic and/or antagonistic effects should be noted [40].

Data related to the impact of F^−^ on trace elements are lacking, and most studies, mainly epidemiological, are conducted on blood, stool, and hair samples [96,97,98]. Since F^−^ exposure disturbs the gut microbiota and leads to dysbiosis, we could assume that one of the toxicity mechanisms is the distorted availability of trace elements [99]. As mentioned previously, systemic F^−^ toxicity studies are central in this research area, and it is essential to establish its complex effects, especially in the gut lumen, to determine the exact mechanism by which absorption of trace elements is altered. While trace elements are essential and present in all cells and tissues of the body (in different forms and concentrations), we also recommend the use of dose–response analysis for more adequate and precise conclusions. Since there is no clear evidence related to F^−^ toxicity in combination with metalloids and metals, further studies are required in order to verify their mutual influences in different cell types.

The BMD5 approach is superior to the NOAEL method, since it calculates the dose that induces a 5% change in effect. We recommend using the BMD method with human environmental studies to help establish fluoride safety limits. When applying BMD results from animal studies to humans, a toxicological point of departure should be determined. This means that animal data provide a moderate toxicological threshold (a lower confidence limit) to ensure safe fluoride concentrations [21]. Follow-up studies should also use physiologically based pharmacokinetic (PBPK) modeling. This will help to extrapolate applicable doses from animals to humans, considering differences in F^−^ metabolism and accumulation among species, including sex-specific differences. The goal of the use of these methods is long-term prediction of possible fluoride toxicity [100].

Notably, in our experiment, the highest F^−^ dose of 150 mg/L exceeded typical environmental exposure levels. However, according to the available data, it has been shown that F^−^ given in this dose subacutely may induce apoptosis, abnormal cytokine expression, oxidative stress and DNA damage [101,102]. Additionally, our preliminary data on the effect of the same concentration of F^−^ demonstrated oxidative stress development and DNA damage. Accordingly, a F^−^ concentration of 150 mg/L was chosen for the design of our study as the highest dose, as it is the established toxic dose in a rat model [29,103,104,105]. Another limitation of the present study is that only male rats were used, in order to avoid the influence of fluctuating estrogen levels. Therefore, the findings of this study are applicable exclusively to male subjects, which leads to limited generalization due to sex differences. The sample size in our research was reduced in accordance with the 3R principle (Replacement, Reduction, Refinement) but relatively low compared to the number of parameters. Further, our study had a short duration (28 days), and subchronic and/or chronic research is required for a more detailed evaluation of the presented parameters, together with other exposure routes besides drinking water. Assessment of liver and kidney biochemical parameters (for instance, bilirubin, ALT, AST, urea, albumin, creatinine, etc.) is desirable for more precise inflammation and damage detection. The absence of behavioral (neurological changes, somatization or cognitive behaviors) or clinical indicators of fluoride toxicity is also one of the limitations of this research. Further, it should be noted that the choice of the exponential and Hill models for BMD5 modeling represents a limitation, as different mathematical models may yield varying BMD5 estimates, potentially affecting the interpretation of dose–response relationships.

## 5. Conclusions

Our findings confirmed the sensitivity of liver and kidney tissues to moderately elevated F^−^ concentrations, reflected in altered BWG, histopathological changes, oxidative stress, and essential element imbalance. Subacute F^−^ exposure induced marked tissue damage, with liver inflammation and fibrosis and kidney impairments ranging from vacuolar degeneration to glomerular necrosis. Fluoride accumulation promoted oxidative stress by increasing O_2_·^−^, TOS, and AOPP levels; reducing SH groups; altering SOD activity; and disrupting Cu, Zn, Fe, and Mn homeostasis, even at low doses. Dose–response modeling showed the lowest liver BMDL_5_ values for Mn (0.1 mg F^−^/L, external dose) and O_2_·^−^ (0.06 mg F^−^/kg, internal dose), while in the kidneys the lowest values were observed for AOPP (6.5 × 10^−6^ mg F^−^/L, external dose) and SOD (1 × 10^−4^ mg F^−^/kg, internal dose). These low BMDL_5_ values indicate that even minimal fluoride doses may cause measurable (5% vs. control) disturbances in redox status and essential element homeostasis, potentially impairing liver and kidney function. This study expands the limited evidence on fluoride-induced liver and kidney toxicity, highlighting the need for updated risk assessments and careful monitoring of fluoride exposure in the context of water fluoridation policies.

## Figures and Tables

**Table 1:** Mean (±SD) BWG for experimental groups of Wistar rats exposed to different fluoride concentrations.

Group	Parameter	Day 7	Day 14	Day 21	Day 28
Control	BWG	0.27 ± 0.03	0.68 ± 0.05 ****	1.01 ± 0.08 ****^##^	0.95 ± 0.10 ****^###^
F 10 mg/L F^−^	BWG	0.25 ± 0.03	0.63 ± 0.07 ****	0.91 ± 0.12 ****^##^	0.97 ± 0.21 ***^##^
	% to control	−7.28%	−7.82%	−9.60%	1.98%
F 25 mg/L F^−^	BWG	0.21 ± 0.03	0.59 ± 0.07 ****	0.93 ± 0.13 ****^###^	0.97 ± 0.21 **^##^
	% to control	−20.86%	−13.71%	−7.49%	1.98%
F 50 mg/L F^−^	BWG	0.14 ± 0.06	0.53 ± 0.09 ****	0.81 ± 0.12 ****^####^	0.86 ± 0.14 ****^###$^
	% to control	−45.90%	−22.13%	−19.46%	−10.45%
F 100 mg/L F^−^	BWG	0.18 ± 0.02	0.54 ± 0.02 ****	0.84 ± 0.05 ****^####^	0.82 ± 0.03 ****^####^
	% to control	−30.20%	−19.90%	−16.41%	−14.11%
F 150 mg/L F^−^	BWG	0.17 ± 0.03	0.59 ± 0.04 ****	0.92 ± 0.08 ****^####^	0.91 ± 0.08 ****^###^
	% to control	−37.11%	−12.27%	−8.61%	−5.16%

** *p* < 0.01; *** *p* < 0.001; **** *p* < 0.0001 (in comparison with the 7th day of the experiment). ^##^ *p* < 0.01; ^###^ *p* < 0.001; ^####^ *p* < 0.0001 (in comparison with the 14th day of the experiment). ^$^ *p* < 0.05 (in comparison with the 21st day of the experiment). Repeated-measures LMM with FDR-adjusted *p*-values.

**Table 2:** Results of the liver histopathological analysis—Knoddel scores.

Group	Periportal and Bridging Necrosis (A)	Intralobular Degeneration and Focal Necrosis (B)	Portal Inflammation (C)	Fibrosis (D)	HAI Knoddel (A + B + C + D)	Interpretation
10 mg/L F^−^	0	0	0	0	0	No inflammation
25 mg/L F^−^	0	0	1	1	2	Minimal inflammation
50 mg/L F^−^	1	1	1	3	6	Mild inflammation
100 mg/L F^−^	3	3	3	3	12	Moderate
150 mg/L F^−^	3	3	4	4	14	Marked

Periportal and bridging necrosis: none (0), mild piecemeal necrosis (1), moderate piecemeal necrosis (3); intralobular degeneration and focal necrosis: none (0), mild (1), moderate (3); portal inflammation: none (0), mild (1), moderate (3), marked (4); fibrosis: none (0), mild (1), moderate (3), marked (4). The final HAI score: no inflammation (0), minimal inflammation (1–4), mild inflammation (5–8), moderate inflammation (9–12), marked inflammation (13–18).

**Table 3:** Results of the kidney tissue histopathological analysis (modified EGTI scoring system) and scoring of kidney cortical damage.

EGTI Scoring						Cortical Damage
Groups	Tubular	Endothelial	Glomerular	Tubular/Interstitial	Sum of the Scores	Scoring
Control	0	0	0.2	0	0.2	0
10 mg/L F^−^	0.2	0	0.2	0	0.4	0
25 mg/L F^−^	0.4	0.4	0.6	0.4	1.8	0.5
50 mg/L F^−^	1.4	1	1.6	0.6	4.6	1
100 mg/L F^−^	1.6	1	1.8	2.6	7.0	1
150 mg/L F^−^	2.6	2.2	2.6	3	10.4	1

EGTI scoring system—tubular: no damage (0); loss of brush border in <25% of tubular cells and preserved integrity of basal membrane (1), loss of brush border in >25% of tubular cells and thickened basal membrane (2), plus inflammation cast formation and necrosis < 60% of tubular cells (3), and necrosis in >60% of tubular cells (4); endothelial: no damage (0), endothelial swelling (1), endothelial disruption (2), or endothelial loss (3); glomerular: no damage (0), thickening of the Bowman capsule (1), glomerular retraction (2), and glomerular fibrosis (3); tubular/interstitial: no damage (0), inflammation and hemorrhage in <25% of tissue (1), plus necrosis in <25% of tissue (2), necrosis < 60% (3), and necrosis > 60% of tubular and interstitial tissue (4). Cortical damage scoring—no damage (0), small focal damaged areas (0.5), <10% cortical damaged zone (1), 10–25% cortical damaged zone (2), 25–75% cortical damaged zone (3), >75% cortical damaged zone (4).

**Table 4:** Essential element levels in liver and kidney tissues of Wistar rats subacutely exposed to increasing F^−^ concentrations.

Tissue	Bioelement	Groups (n = 5)					
		Control	10 mg/L F^−^	25 mg/L F^−^	50 mg/L F^−^	100 mg/L F^−^	150 mg/L F^−^
Liver	Cu (μg/g)	3.73 3.37–4.22	3.41 3.36–3.84	3.74 3.42–5.02	3.67 3.37–3.77	3.14 * 2.99–3.34	2.99 ** 2.93–3.25
	Zn (μg/g)	29.86 28.79–31.93	31.18 29.96–31.84	30.29 29.73–33.41	39.57 ** 33.59–58.39	36.06 * 31.93–40.54	31.75 30.97–54.93
	Fe (μg/g)	122.9 ± 19.59	129.40 ± 9.29	120.50 ± 2.87	134.00 ± 13.49	120.3 ± 16.23	121.80 ± 7.67
	Mn (μg/g)	3.09 ± 0.95	3.12 ± 0.82	3.48 ± 1.30	4.15 ± 0.71	4.40 ± 0.59 *	4.43 ± 1.09 *
Kidneys	Cu (μg/g)	7.13 ± 1.78	8.1 ± 1.44	7.74 ± 2.03	8.55 ± 2.65	6.77 ± 2.31	5.48 ± 1.84
	Zn (μg/g)	113.40 74.5–143.9	141.8 85.27–159.90	138.7 69.43–225.80	199.70 152.1–343.3	510.1 * 87.72–560.60	43.87 23.21–66.48
	Fe (μg/g)	85.65 ± 15.70	91.06 ± 11.95	74.87 ± 18.26	65.57 ± 7.21 *	57.87 ± 21.33 **	35.96 ± 4.07 ****
	Mn (μg/g)	20.59 ± 5.73	20.63 ± 5.88	15.34 ± 8.46	9.73 ± 6.30 **	4.60 ± 1.66 ***	2.87 ± 3.73 ***

Cu copper; Zn, zinc; Fe, iron; Mn, manganese. One-way ANOVA followed by Fisher’s least significant difference (LSD) and Kruskal–Wallis tests, followed by Dunn’s post hoc test. * *p* < 0.05; ** *p* < 0.01; *** *p* < 0.001; **** *p* < 0.0001 (compared with the control group). Results are given as means ± SDs or as the medians and interquartile ranges (25–75%).

**Table 5:** External and internal dose-dependent fluoride response in liver tissue.

External Dose								
Parameter	Fitted Models	Conv.	Loglik.	No. Par.	AIC	BMDL5 (mg F^−^/L)	BMDU5 (mg F^−^/L)	Trend
Cu	Full model	1	25.98	7	−37.96	/	/	
	Null model	1	16.45	2	−28.9	/	/	
	Expon. m3-	1	23	4	−38	29.8	129	+ ↓
	Expon. m5-	1	23.53	5	−37.06	/	/	
	Hill m3-	1	23.01	4	−38.02	30.1	129	+ ↓
	Hill m5-	1	23.8	5	−37.6	/	/	
Zn	Full model	1	6.1	7	1.8	/	/	
	Null model	1	1.02	2	1.96	/	/	
	Expon. 3-	1	3.35	4	1–3	/	/	
	Expon. 5-	1	5.06	5	−0.12	7.3	42.5	+ ↑
	Hill m3-	1	3.35	4	1.3	/	/	
	Hill m5-	1	4.47	5	1.06	/	/	
Mn	Full model	1	1.55	7	10.9	/	/	
	Null model	1	−4.56	2	13.12	/	/	
	Expon. 3-	1	0.62	4	6.76	0.05	61.5	+ ↑
	Expon. 5-	1	1.55	5	6.9	/	/	
	Hill m3-	1	0.63	4	6.74	0.1	61.2	+ ↑
	Hill m5-	1	1.53	5	6.94	/	/	
SH	Full model	1	3.67	7	6.66	/	/	
	Null model	1	−2.89	2	9.78	/	/	
	Expon. 3-	1	1.91	4	4.18	/	/	
	Expon. 5-	1	3.07	5	3.86	10.8	73.1	+ ↓
	Hill m3-	1	1.91	4	4.18	0.8	95.4	+ ↓
	Hill m5-	1	2.81	5	4.38	/	/	
MDA	Full model	1	3.78	7	6.44	/	/	
	Null model	1	−2.82	2	9.64	/	/	
	Expon. 3-	1	2.19	4	3.62	/	/	
	Expon. 5-	1	3.27	5	3.46	9.6	74.4	+ ↓
	Hill m3-	1	2.19	4	3.62	1.1	94.1	+ ↓
	Hill m5-	1	3.04	5	3.92	/	/	
**Internal Dose**								
**Parameter**	**Fitted Models**	**Conv.**	**Loglik.**	**No. Par.**	**AIC**	**BMDL** **(mg F^−^/kg)**	**BMDU** **(mg F^−^/kg)**	**Trend**
O_2_·^−^	Full model	/	/	/	/	/	/	
	Null model	1	8.4	2	−12.8	/	/	
	Expon. 3-	1	11.76	4	−15.52	/	/	
	Expon. 5-	1	13.55	5	−17.1	0.06	0.2	+ ↑
	Hill m3-	1	11.79	4	−15.58	/	/	
	Hill m5-	0	13.46	5	−16.92	0.03	0.3	+ ↑

AIC, Akaike information criterion; BMDL5, lower confidence limit of the benchmark dose; BMDU5, upper confidence limit of the benchmark dose; Cu, copper; Loglik, loglikelihood; MDA, malondialdehyde; Mn, manganese; No. Par., number of parameters; O_2_·^−^, superoxide anions; SH, total thiol groups; Zn, zinc. ↑ positive trend; ↓ negative trend.

**Table 6:** External and internal dose-dependent fluoride response in kidney tissue.

External Dose								
Parameter	Fitted Models	Conv.	Loglik.	No. Par.	AIC	BMDL5 (mg F^−^/L)	BMDU5 (mg F^−^/L)	Trend
Cu	Full model	1	−1.63	7	17.26	/	/	
	Null model	1	−6.06	2	16.12	/	/	
	Expon. m3-	1	−2.42	4	12.84	28.1	137	+ ↓
	Exp. m5-	1	−2.28	5	14.56	/	/	
	Hill m3-	1	−2.42	4	12.84	28.1	133	+ ↓
	Hill m5-	1	−2.36	5	14.72	/	/	
Zn	Full model	1	−30.74	7	75.48	/	/	
	Null model	1	−38.91	2	81.82	/	/	
	Expon. 3-	1	−34.07	4	76.14	63.2	104	+ ↓
	Expon. 5-	1	−34.09	5	78.18	/	/	
	Hill m3-	1	−34.99	4	77.98	47.8	93.1	+ ↓
	Hill m5-	1	−35.07	5	80.14	/	/	
Fe	Full model	1	6.67	7	0.66	/	/	
	Null model	1	−12.42	2	28.84	/	/	
	Expon. 3-	1	5.45	4	−2.9	7.4	58.8	+ ↓
	Expon. 5-	1	5.44	5	−0.88	/	/	
	Hill m3-	1	5.44	4	−2.88	7.5	58.9	+ ↓
	Hill m5-	0	5.41	5	−0.82	/	/	
Mn	Full model	1	−39.84	7	93.68	/	/	
	Null model	1	−51.97	2	107.94	/	/	
	Expon. 3-	1	−40.33	4	88.66	83.1	149	+ ↓
	Expon. 5-	1	−40.38	5	90.76	/	/	
	Hill m3-	1	−40.35	4	88.7	83.1	149	+ ↓
	Hill m5-	1	−40.44	5	90.88	/	/	
SOD	Full model	/	3.38	7	7.24	/	/	
	Null model	1	−6.7	2	17.4	/	/	
	Expon. 3-	1	0.78	4	6.44	42.1	118	+ ↑
	Expon. 5-	1	0.77	5	8.46	/	/	
	Hill m3-	1	0.46	4	7.08	31.1	105	+ ↑
	Hill m5-	1	0.44	5	9.12	/	/	
SH	Full model	1	−5.36	7	24.72	/	/	
	Null model	1	−13.49	2	30.98	/	/	
	Expon. m3-	1	−6.24	4	20.48	0.002	13.4	+ ↓
	Expon. m5-	1	−5.55	5	21.1	/	/	
	Hill m3-	1	−6.24	4	20.48	0.3	13.4	+ ↓
	Hill m5-	1	−5.48	5	20.96	/	/	
AOPP	Full model	1	−5.96	7	25.92	/	/	
	Null model	1	−13.48	2	30.96	/	/	
	Expon. 3-	1	−6.25	4	20.5	0.001	9.9	+ ↑
	Expon. 5-	1	−6.25	5	22.5	/	/	
	Hill m3-	1	−6.25	4	20.5	6.5 × 10^−6^	9.9	+ ↑
	Hill m5-	1	−6.25	5	22.5	/	/	
**Internal Dose**								
**Parameter**	**Fitted Models**	**Conv.**	**Loglik.**	**No. Par.**	**AIC**	**BMDL5** **(mg F^−^/kg)**	**BMDU5** **(mg F^−^/kg)**	**Trend**
Cu	Full model	/	/	/	/	/	/	
	Null model	1	3.05	2	−2.1	/	/	
	Expon. 3-	1	5.93	4	−3.86	/	/	
	Expon. 5-	1	7.35	5	−4.7	1.3	1.8	+ ↑
	Hill m3-	1	5.95	4	−3.9	/	/	
	Hill m5-	1	7.02	5	−4	/	/	
Zn	Full model	/	/	/	/	/	/	
	Null model	1	−5.98	2	15.96	/	/	
	Expon. 3-	1	3.41	4	1.18	/	/	
	Expon. 5-	1	3.41	5	3.18	2.48 × 10^−6^	0.6	+ ↑
	Hill m3-	1	3.41	4	1.18	0.05	0.6	+ ↑
	Hill m5-	1	3.4	5	3.2	/	/	
SOD	Full model	/	/	/	/	/	/	
	Null model	1	0.33	2	3.34	/	/	
	Expon. 3-	1	2.27	4	3.46	/	/	
	Expon. 5-	1	5.32	5	−0.6	1 × 10^−4^	1.4	+ ↓
	Hill m3-	1	2.29	4	3.42	/	/	
	Hill m5-	1	5.1	5	−0.2	0.005	0.9	+ ↓

AIC, Akaike information criterion; AOPP, advanced oxidation protein product level; BMDL5, lower confidence limit of the benchmark dose; BMDU5, upper confidence limit of the benchmark dose; Cu, copper; Fe, iron; Loglik, loglikelihood; MDA, malondialdehyde; Mn, manganese; No. Par., number of parameters; O_2_·^−^, superoxide anions; SH, total thiol groups; SOD, superoxide dismutase activity; Zn, zinc. ↑ positive trend; ↓ negative trend.

## Data Availability

The data presented in this study are available on request from the corresponding author due to research privacy.

## Supplementary Materials

The following supporting information can be downloaded at: https://www.mdpi.com/article/10.3390/jox16020063/s1, Figure S1: Liver, control group without pathological changes, 400×; Figure S2: Liver, 50 mg/L F^−^, congested blood vessels, apoptotic hepatocytes with cytoplasmic and nuclear condensation, 400×; Figure S3: Liver, 100 mg/L F^−^, focal necrosis, 400×; Figure S4: Liver, 100 mg/L F^−^, portal—portal bridging necrosis, apoptotic hepatocytes, 200×; Figure S5: Liver, 150 mg/L F^−^, portal—central bridging necrosis, apoptosis of hepatocytes with cytoplasmic and nuclear condensation and nuclear fragmentation, 200×; Figure S6: Liver, 150 mg/L F^−^, slight portal fibrosis, 200×; Figure S7: Kidney, control group without pathological changes, 400×; Figure S8: Kidney, 50 mg/L F^−^, 400×; Figure S9: Kidney, 100 mg/L F^−^, 400×; Figure S10: Kidney, 150 mg/L F^−^ 400×; Figure S11: Kidney, 150 mg/L F^−^ 400×; Figure S12: Kidney, 150 mg/L F^−^ 400×.

## Abbreviations

AOPP	Advanced oxidation protein product level
BMD	Benchmark dose
BMDL5	BMD lower interval
BMDU5	BMD upper interval
BMR	Benchmark response
BWG	Bodyweight gain
EFSA	European Food Safety Authority European Food Safety Authority
F	Fluoride
FDR	False discovery rate
HAI	Histological activity index
HCIO_4_	Perchloric acid
HNO_3_	Nitric acid
LMM	Linear mixed model
MDA	Malondialdehyde
NaF	Sodium fluoride
NOAEL	No-observed-adverse-effect level
O_2_·^−^	Superoxide anion
OS	Oxidative stress
ROS	Reactive oxidative species
SH	Total thiol groups
SOD1	Superoxide dismutase activity
TOS	Total oxidative status
