# Supplementary material for: Benchmark Approach to Unravel Fluoride Toxicity: Liver and Kidney Disruptions in Subacutely Exposed Rats
Source: J Xenobiot. 2026 Apr 7;16(2):63. doi: 10.3390/jox16020063 (PMC13117112; doi:10.3390/jox16020063)

Journal of Xenobiotics

Supplementary Materials: Benchmark approach to unravel fluoride toxicity: liver and kidney disruptions in subacutely exposed rats

Jelena Radovanović, Sanja Milutinović-Smiljanić, Biljana Antonijević, Katarina Baralić, Marijana Ćurčić, Đurđica Marić, Zoran Mandinić

Figure S1: Liver, control group without pathological changes, 400x.

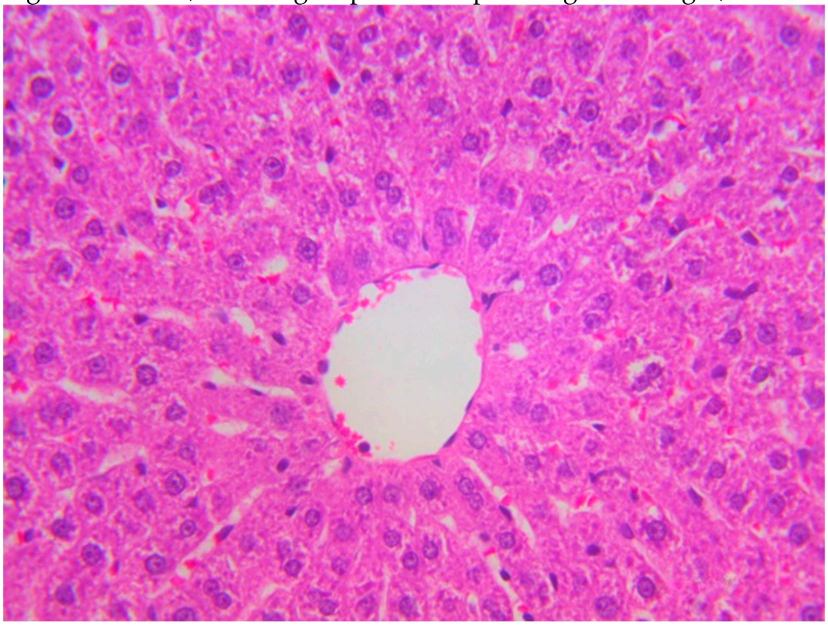

Figure S2: Liver, 50 mg/L F−, congested blood vessels, apoptotic hepatocytes with cytoplasmic and nuclear condensation, 400x.

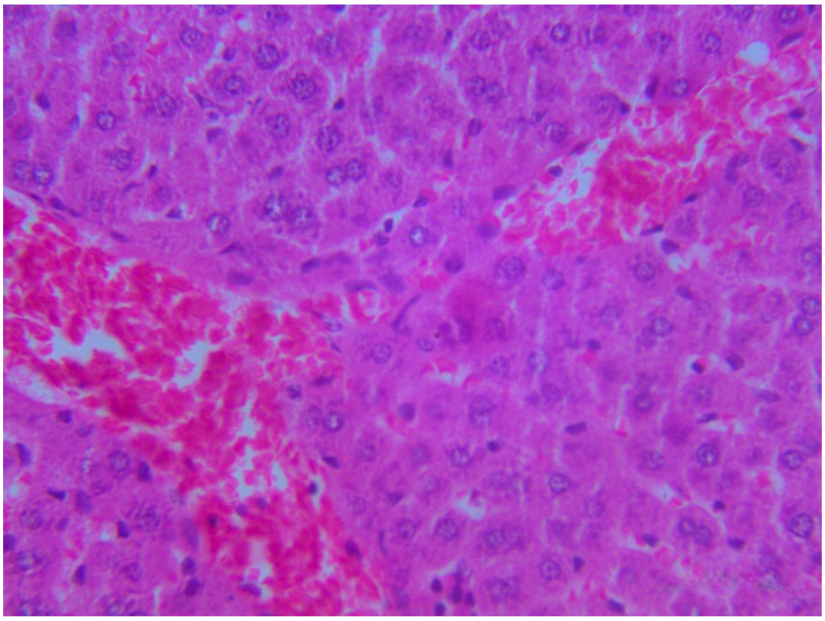

Figure S3: Liver, 100 mg/L F−, focal necrosis, 400x.

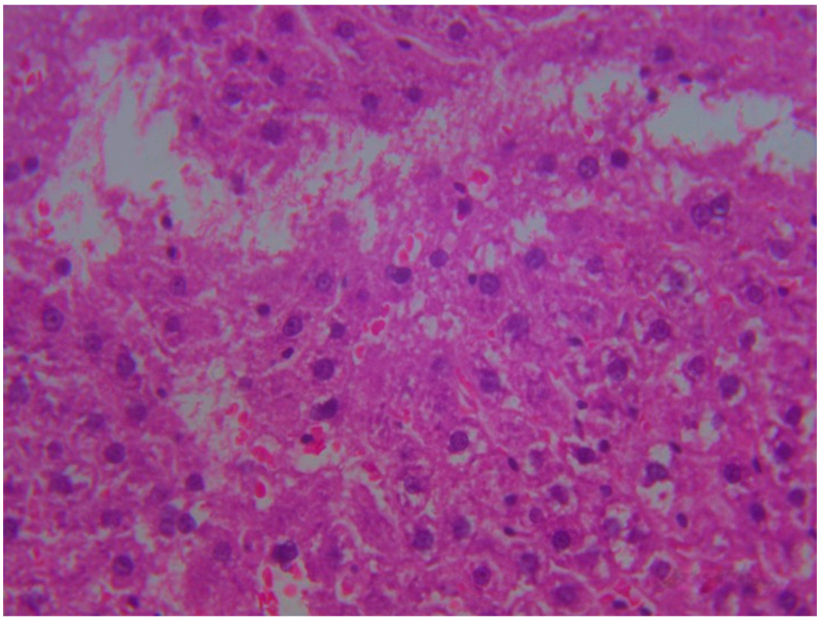

Figure S4: Liver, 100 mg/L F−, portal—portal bridging necrosis, apoptotic hepatocytes, 200x.

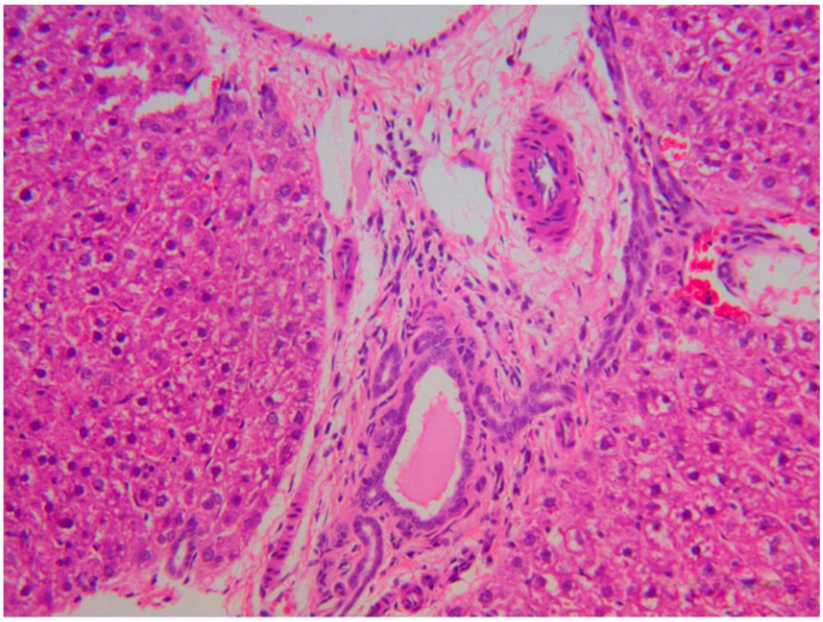

Figure S5: Liver, 150 mg/L F−, portal–-central bridging necrosis, apoptosis of hepatocytes with cytoplasmic and nuclear condensation and nuclear fragmentation, 200x.

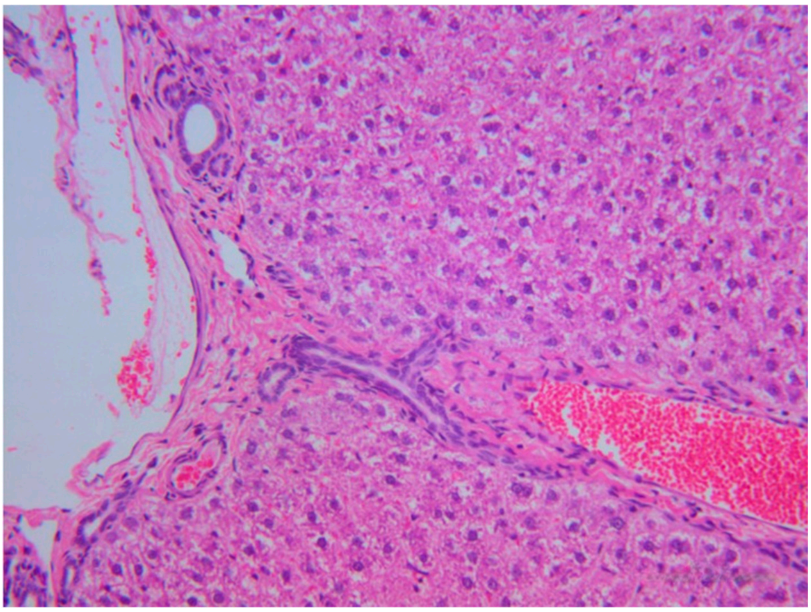

Figure S6: Liver, 150 mg/L F−, slight portal fibrosis, 200x

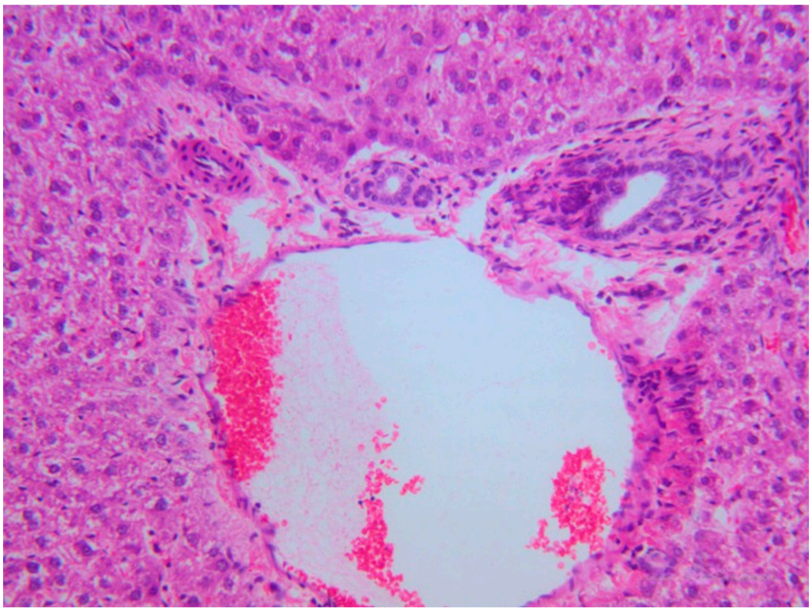

Figure S7: Kidney, control group without pathological changes, 400x.

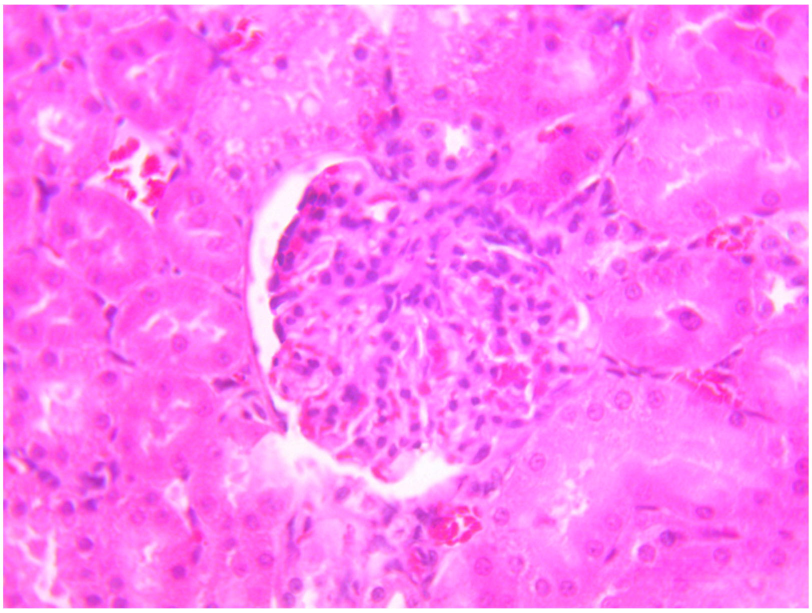

Figure S8: Kidney, 50 mg/L F−, 400x.

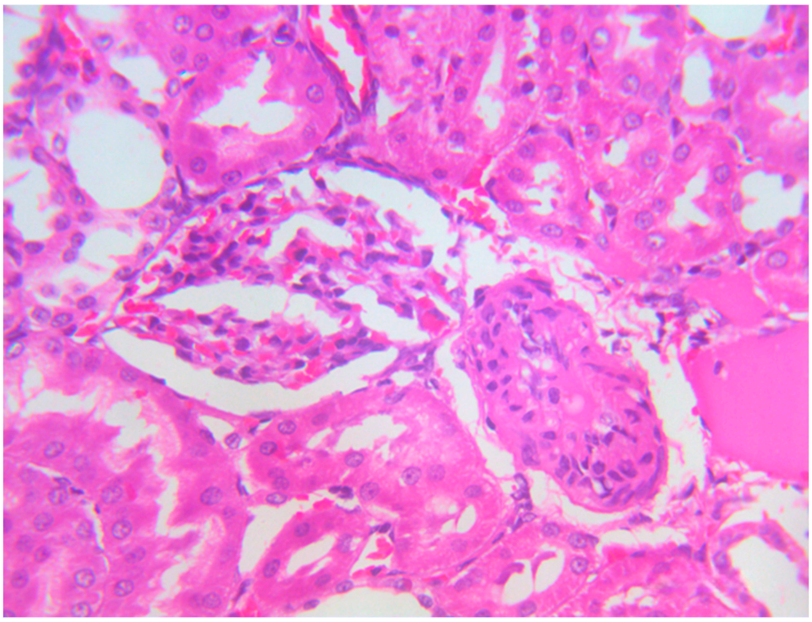

Figure S9: Kidney, 100 mg/L F−, 400x.

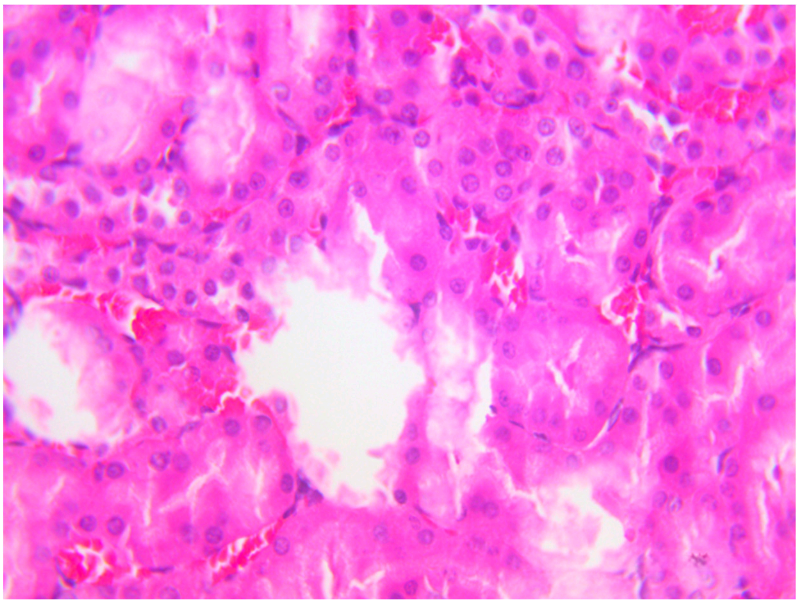

Figure S10: Kidney, 150 mg/L F− 400x.

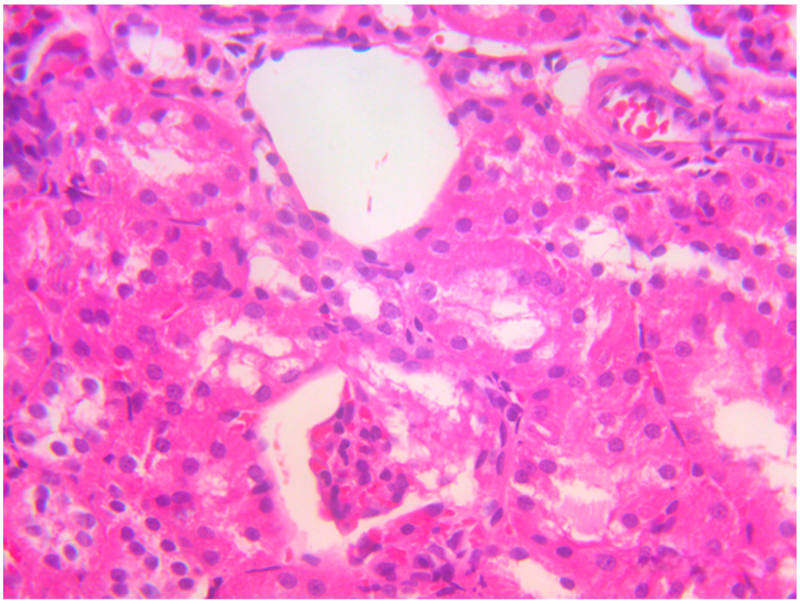

Figure S11: Kidney, 150 mg/L F− 400x.

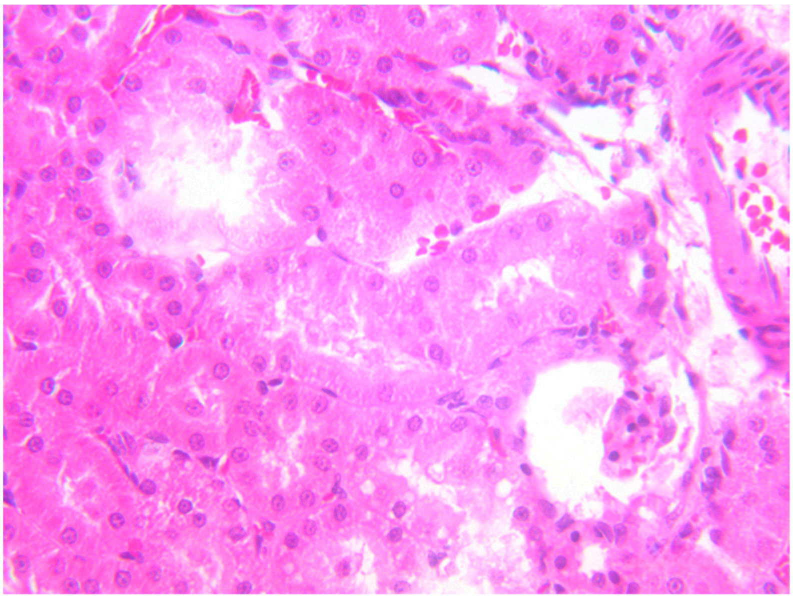

Figure S12: Kidney, 150 mg/L F− 400x.

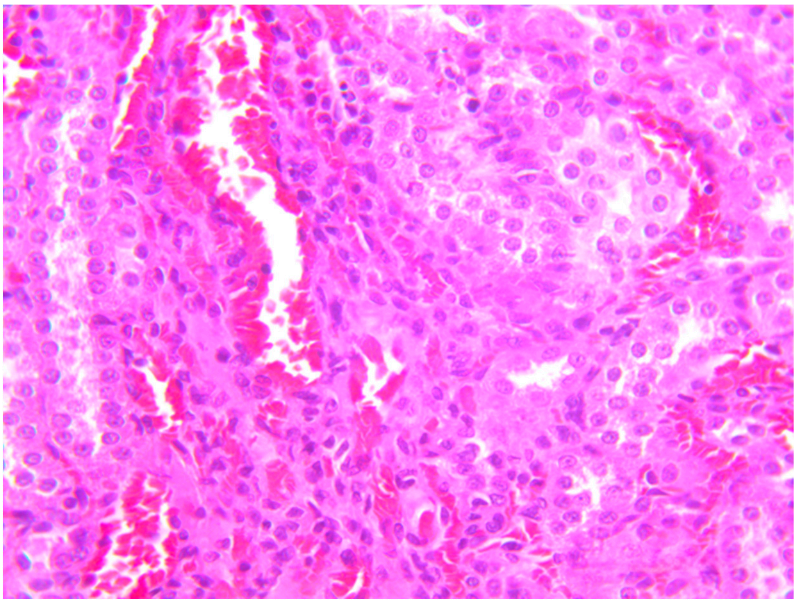

Supplement: Supplementary file 1 [file jox-16-00063-s001.zip › jox-4181202-supplementary.pdf]
